# In Vivo Imaging of Prostate Cancer Tumors and Metastasis Using Non-Specific Fluorescent Nanoparticles in Mice

**DOI:** 10.3390/ijms18122584

**Published:** 2017-12-01

**Authors:** Coralie Genevois, Arnaud Hocquelet, Claire Mazzocco, Emilie Rustique, Franck Couillaud, Nicolas Grenier

**Affiliations:** 1 Imagerie Moléculaire et Thérapies Innovantes en Oncologie, IMOTION, EA 7435, Bordeaux University, F33076 Bordeaux, France; coralie.genevois@u-bordeaux.fr (C.G.); arnaud.hocquelet@gmail.com (A.H.); cmazzocco@immusmol.com (C.M.); 2 CEA Grenoble, LETI-DTBS, MINATEC Campus, F38054 Grenoble, France; Emilie.RUSTIQUE@cea.fr

**Keywords:** prostate cancer, fluorescence imaging, bioluminescence imaging, fluorescence tomography, enhanced permeability and retention (EPR) effect, LipImage^TM^

## Abstract

With the growing interest in the use of nanoparticles (NPs) in nanomedicine, there is a crucial need for imaging and targeted therapies to determine NP distribution in the body after systemic administration, and to achieve strong accumulation in tumors with low background in other tissues. Accumulation of NPs in tumors results from different mechanisms, and appears extremely heterogeneous in mice models and rather limited in humans. Developing new tumor models in mice, with their low spontaneous NP accumulation, is thus necessary for screening imaging probes and for testing new targeting strategies. In the present work, accumulation of LipImage^TM^ 815, a non-specific nanosized fluorescent imaging agent, was compared in subcutaneous, orthotopic and metastatic tumors of RM1 cells (murine prostate cancer cell line) by in vivo and ex vivo fluorescence imaging techniques. LipImage^TM^ 815 mainly accumulated in liver at 24 h but also in orthotopic tumors. Limited accumulation occurred in subcutaneous tumors, and very low fluorescence was detected in metastasis. Altogether, these different tumor models in mice offered a wide range of NP accumulation levels, and a panel of in vivo models that may be useful to further challenge NP targeting properties.

## 1. Introduction

New probes for tumor imaging and local therapies are an important clinical need. Currently, clinical developments are mainly based on new radionuclides for positron emission tomography (PET). However, there is growing interest in nanoparticles (NPs), because they offer various specific properties, including high surface-to-volume ratio, high surface energy, and a wide range of additional mechanical, thermal, electrical, magnetic, and optical properties [1,2,3]. NPs further offer possibilities to combine several contrast agents for multimodal imaging, to be decorated with various biological and chemical moieties and to cargo therapeutic agents.

Determining the distribution of nanocarriers within the body following systemic administration in order to achieve high accumulation of NPs in tumors with low background in other tissues is the major challenge for nanomedicine. NP characteristics have an impact on their pharmacokinetics [4,5], and longer plasmatic half-life favors higher accumulation within the tumor. Such accumulation of NPs in tumors results from different mechanisms, either specific or nonspecific, and involves different cell populations, including cancer and stroma cells [6,7].

The enhanced permeability and retention (EPR) effect [8,9] has been suggested to be the major underlying mechanism of passive NP accumulation in tumors. The EPR effect, although efficient in mice, appears to be extremely heterogeneous—or possibly totally ineffective—in humans [10,11], resulting in low or no accumulation of NPs in human tumors. Since the EPR effect fails in the clinic, new tumor models with low spontaneous NP accumulation are required to screen imaging probes and to test new targeting strategies.

Fluorescent imaging on mice models is a convenient way to initiate the screening process of NP-based imaging probes and to provide key information about NP properties. Although in vivo fluorescence imaging does not discriminate between the different mechanisms involved in NP accumulation in tumors, it allows for rapid evaluation of the overall targeting efficiency [12]. In order to develop new tumor models in mice, the aim of the present work is to study NP accumulation for a single tumor cell line according to different tumor locations. For this purpose, RM1, a murine prostate cancer cell line, was used at different implantation sites to generate subcutaneous, orthotopic and metastatic tumors. LipImage^TM^ 815, a non-specific nanosized (80-nm diameter) fluorescent imaging agent, was injected intravenously to compare NP accumulation in the various tumor locations and types.

## 2. Results

### 2.1. In Vivo Imaging of LipImage^TM^ 815 in Mice Bearing RM1-Subcutaneous Tumors

**Figure 1:**
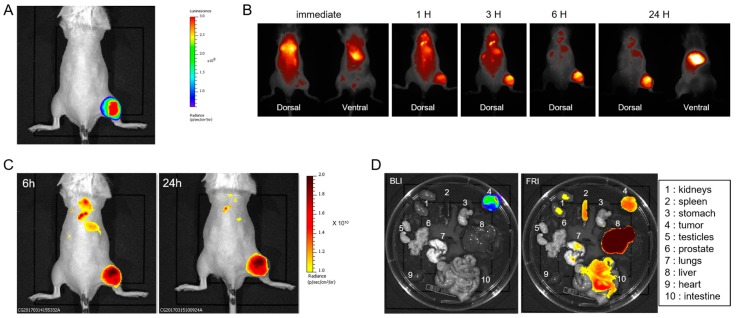
In vivo detection of LipImage^TM^ 815 accumulation in subcutaneous tumors. (**A**) Bioluminescence image (BLI) of a representative mouse; (**B**) Fluorescence reflectance imaging (FRI) of a representative mouse at different time after LipImage^TM^ 815 injection; (**C**) FRI of a representative mouse 6 h and 24 h after LipImage^TM^ 815 injection, respectively; (**D**) Ex vivo BLI and FRI of mouse organs 24 h after LipImage^TM^ 815 injection.

RM1-CMV/Fluc cells were injected subcutaneously (2 × 10^6^ cells/100 µL) into the posterior right leg of the mice (*n* = 3). One week after injection of cells, tumors were monitored by bioluminescence imaging (BLI) (Figure 1A). LipImage^TM^ 815 was then injected into the mice (14 × 10^12^ particles) via the tail vein and could be monitored by live fluorescence imaging (Figure 1B and Video S1).

The fluorescent signal was immediately detectable in vasculature, and within a few seconds in the kidney. Hyper-vascularization revealed by instant fluorescence reflectance imaging (FRI) was the first indication of the presence of a tumor on the right leg (Video S1). After 1 h, (Figure 1B), the fluorescent signal had accumulated in the liver and in the tumor. At 6 h and 24 h, both instant FRI (Figure 1B) and dark box FRI (Figure 1C) revealed a high fluorescent signal resulting from LipImage^TM^ 815 accumulation in the subcutaneous tumors and in the liver, with limited background fluorescence in other tissues. As shown in Figure S1, LipImage^TM^ 815 accumulation did not interfere with the BLI signal, which increased as the tumor grew. Mice were euthanized 24 h after LipImage^TM^ 815 injection, and organs were imaged by BLI and FRI (Figure 1D). BLI revealed the presence of tumor cells exclusively in the tumor sample. Quantification of FRI signal for ex vivo samples is expressed as photons·s^−1^·cm^−2^·sr^−1^. The highest fluorescent signal from LipImage^TM^ 815 was found in the liver (1.47 × 10^10^ ± 3.14 × 10^9^ ph·s^−1^·cm^−2^·sr^−1^; *n* = 3). Lower levels were detected in the tumor (4.55 × 10^9^ ± 4.22 × 10^8^ ph·s^−1^·cm^−2^·sr^−1^; *n* = 3), but also in the intestines, kidneys, lungs and spleen. 

### 2.2. In Vivo Imaging of LipImage^TM^ 815 in Mice Bearing RM1-Prostate Tumors

**Figure 2:**
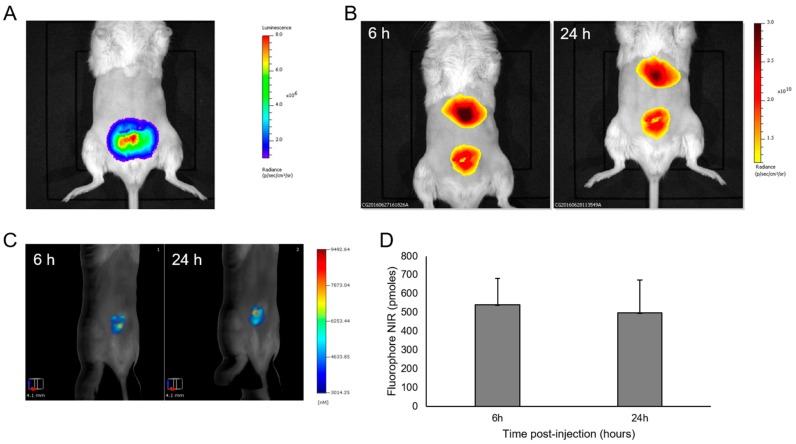
In vivo detection of LipImage^TM^ 815 accumulation in orthotopic prostate tumors. (**A**) Bioluminescence image of a representative mouse; (**B**) FRI and (**C**) FMT of a representative mouse 6 h and 24 h after LipImage^TM^ 815 injection; (**D**) FMT-based quantification of LipImage^TM^ 815 accumulation in the prostate tumor. Mean ± standard deviation.

**Figure 3:**
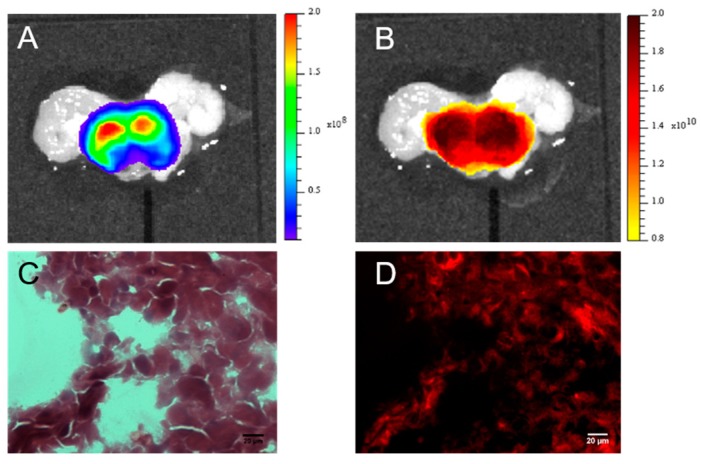
Ex vivo imaging and histology of LipImage^TM^ 815 accumulation in prostate tumors. Prostates were excised and imaged (**A**) by BLI and (**B**) by FRI; (**C**) HES coloration revealed tumor cells and (**D**) epifluorescence revealed fluorescent signals from LipImage^TM^ 815 in prostate cancer cryosection.

RM1-CMV/Fluc cells (5 × 10^5^/10 µL per lobe) were injected into prostate lobes during open surgery (*n* = 5). Tumor growth was monitored by BLI (Figure 2A). Four days after surgery, LipImage^TM^ 815 was injected into the mice (14 × 10^12^ particles) via the tail vein, and the fluorescence signal was followed by FRI and Fluorescence molecular tomography (FMT) 6 and 24 h after injection. As shown in Figure 2B, FRI revealed a fluorescence signal on the ventral face in regions corresponding to the location of the liver and prostate. FMT provided 3D images of the fluorescent signals in the prostate (Figure 2C) and allowed quantification (Figure 2D). Mice were sacrificed 24 h after LipImage^TM^ 815 injection, and the excised prostates were immediately imaged by BLI (Figure 3A) and FRI (Figure 3B). The fluorescent signal from LipImage^TM^ 815 corresponded to the BLI signal from RM1 cells. The fluorescence signal from LipImage^TM^ 815 in the RM1 tumors was quantified (1.65 × 10^10^ ± 4.22 × 10^8^ ph·s^−1^·cm^−2^·sr^−1^; *n* = 5). Prostates were further processed for histology. Hematoxylin-eosin-safran (HES) staining (Figure 3C) revealed cancer cells and NIR microscopy (Figure 3D) revealed the presence of the NIR fluorophore.

### 2.3. In Vivo Imaging of Metastasis with LipImage^TM^ 815

**Figure 4:**
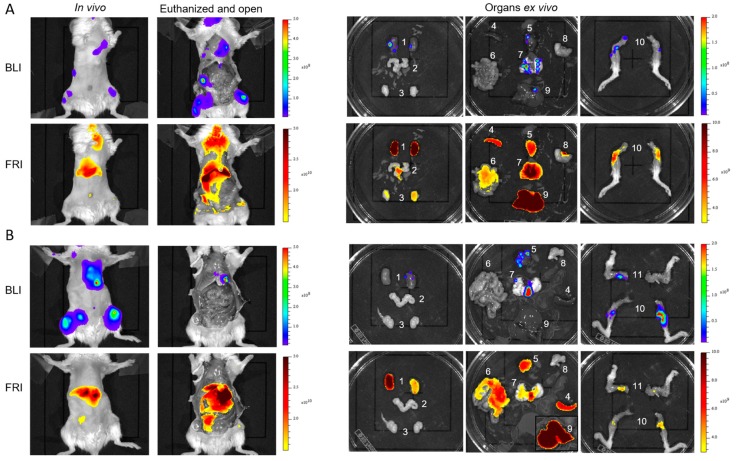
In vivo detection of metastasis. BLI and FRI after LipImage^TM^ 815 injection of 2 representative mice bearing metastatic tumors. Images were taken (**A**) 6 h or (**B**) 24 h after LipImage^TM^ 815 injection first in vivo (column 1), then in euthanized and open mice (column 2). Individual organs were removed and observed by BLI and FRI (columns 3–5). 1: kidneys, 2: prostate, 3: testicles, 4: splenic tumor, 5: heart, 6: intestines, 7: lungs, 8: stomach, 9: liver, 10: posterior legs, 11: anterior legs.

Intra-cardiac echography-guided injection of RM1-CMV/Fluc cells (10^5^/100 µL) in the left ventricle (Video S2) resulted in metastatic dissemination. First metastasis was able to be detected by BLI as soon as 7 days after cells injection (Figure S3; but in the present work, mice were assayed 13 days after injection (Figure 4A,B) (*n* = 11). Six (Figure 4A; *n* = 6) and 24 h (Figure 4B; *n* = 5) after LipImage^TM^ 815 intravenous injection, the main fluorescence location detected was in the liver, but other locations were also detected without a consistent fit with BLI metastasis location (Figure 4A,B, 1st column).

**Figure 5:**
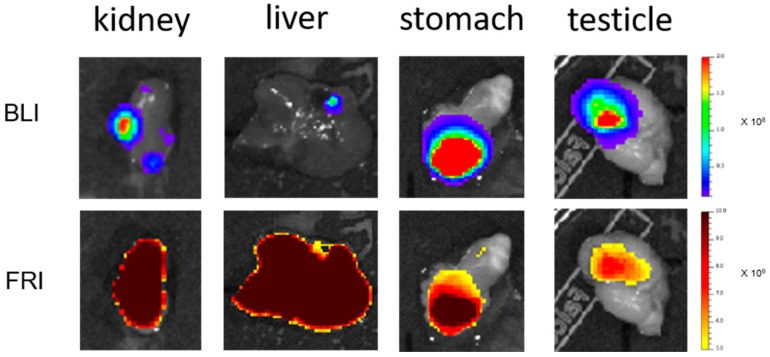
Ex vivo imaging of LipImage^TM^ 815 accumulation in organs containing metastasis. BLI revealed the tumor location and FRI revealed LipImage^TM^ 815 accumulation 6 h or 24 h after intravenous injection. In the kidneys and liver, LipImage^TM^ 815 resulted in a disperse fluorescence signal without detectable accumulation in the metastasis; while in the stomach and testicles, fluorescence coincided with metastasis location. The FRI display scale is identical for all images.

Mice were sacrificed 6 or 24 h after LipImage^TM^ 815 injection. The peritoneal cavity was opened and the skin was removed before BLI and FRI. Again, the liver remained the most fluorescent organ detected (Figure 4A,B, 2nd column). Organs and regions exhibiting a BLI signal were dissected and imaged for BLI and FRI (Figure 4A,B, columns 3–5). Apart from the legs (bone metastasis), the fluorescent signal of NPs did not match the metastatic locations as revealed by BLI. As shown on Figure 5, organs such as the liver and kidneys exhibited high background fluorescence signals throughout the entire organ, and did not exhibit enhancement related to the location of cancer cells. For other organs, such as the stomach or testicles, the fluorescence background was low, and hot spots of fluorescent signal perfectly fitted with metastatic locations revealed by BLI. Nevertheless, the fluorescence level in metastasis remained low, irrespective of location, nearing the background level at 2.31 × 10^9^ ± 1.44 × 10^9^ ph·s^−1^·cm^−2^·sr^−1^ (*n* = 11).

### 2.4. Ex Vivo Quantification of LipImage^TM^ 815 Accumulation in Tumors and Organs

**Figure 6:**
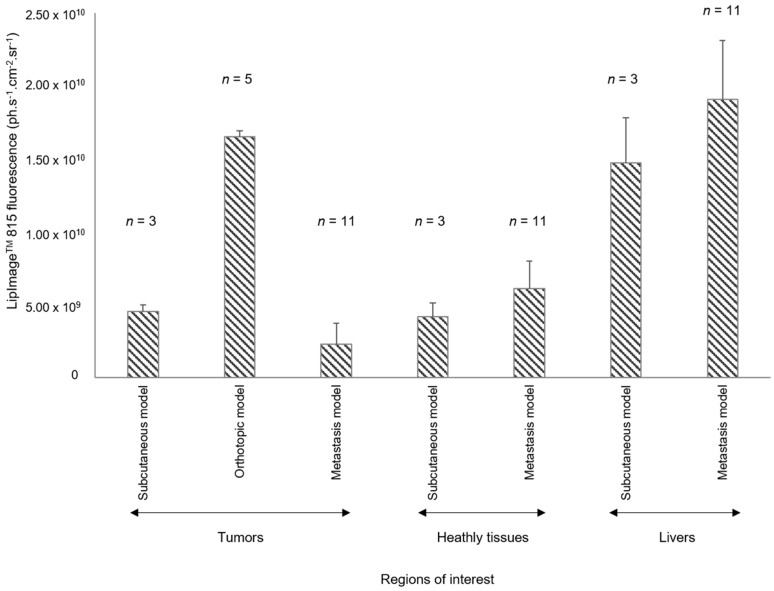
Quantification of fluorescent signal in organs ex vivo, 24 h after intravenous LipImage^TM^ 815 injection. Mean ± standard deviation, (*n*) = number of mice.

Quantification of fluorescence signals in tumors and organs from mice bearing subcutaneous tumors, orthotopic tumors, or metastasis was assessed by FRI ex vivo and the data plotted (Figure 6). The highest fluorescence level from LipImage^TM^ 815 at 24 h was found in healthy livers and orthotopic tumors. The fluorescence level in orthotopic tumors was very high compared with subcutaneous tumors, which was, in turn, higher than metastasis. The fluorescence level in metastasis was very low; lower than the fluorescence level in healthy organs.

## 3. Discussion

Using a single murine prostate cancer cell line (RM1), via different routes, to generate various types of tumors (orthotopic, subcutaneous and metastatic), a constant dose of NPs and a wide panel of in vivo and ex vivo fluorescence imaging techniques, we demonstrated here that the non-specific labeling pattern varied according to tumor type. LipImage^TM^ 815 accumulation was highly predominant in orthotopic tumors when compared with subcutaneous tumors and disseminated metastasis.

RM1 is a mouse prostate cancer cell line syngenic in C57BL/6 mice [13]. It was genetically modified to produce a constitutive Fluc reporter gene and to make in vivo detection by BLI possible, even for small or deep tumors in mice. The BLI signal was not only used to follow tumor growth, but also to compare BLI and fluorescence imaging patterns. As luciferase activity requires oxygen and intraperitoneal injected luciferin (M_W_ = 280 D) as a substrate, the BLI signal further confirms that the tumors are not hypoxic, and are properly vascularized. RM1 tumors are very fast-growing, providing significant orthotopic prostate tumors in 4 days, subcutaneous tumors in 7 days, and metastasis in 13 days. Thus, the present RM1 models provided a complete panel of different tumor types exhibiting different levels of NP accumulation, making wide screening of NPs possible in a short period of time. On the other hand, the rapid growth of RM1 tumors is not favorable for long term studies, as tumors rapidly reached ethical endpoints. Finally, as RM1 is syngenic in C57BL/6 mice, the tumors grew in a microenvironment more representative of the clinical physio-pathological context. The rapid growth of RM1 tumors is a convenient feature for experimental purposes, but clearly diverges from a human tumor growth rate that may influence tumor properties. In the current work, however, the level of NP accumulation in RM1 tumors is not correlated with growing time.

Present data showed that the level of NP accumulation in a tumor was not a characteristic of the tumor cells, but involved other parameters, including tumor location. As a limitation of the present study, the different tumor locations required different implantation methods, which may influence NP accumulation. For metastasis, however, the fluorescence level is low, irrespective of location.

The fluorescent signal in tumors may result from several mechanisms, including retention in extracellular space, binding to or internalization in tumor cells, and tumor microenvironment components [6,7]. Variations in NP accumulation in tumors are often attributed to variations in EPR effect. The EPR effect results from hyper-permeability of the tumor blood vessels and dysfunction of intra-tumoral lymphatics drainage; the first mechanism enables nanoparticles to enter the tumor interstitial space, while the second one allows particles to stay in the tumor for a longer time [14]. In the present work, solid evidence that accumulation results from a true EPR effect is lacking. Vascularization is a key parameter for NP accumulation in different tumors types; but in the present work, no information—such as pericytes coverage or fenestration sizes—is available. Regardless of the mechanism involved, our results confirmed that the resulting non-specific accumulation of NPs in tumors is highly variable. Conversely, accumulation found in some other normal tissues demonstrates that these mechanisms are no more efficient within metastasis or subcutaneous tumors than in healthy tissues.

LipImage^TM^ 815 NPs (50 nm) have previously been characterized for their optical and pharmacokinetic characteristics [15], and have been shown to display a long shelf life, as well as colloidal and optical stabilities, with high brightness and strong and long accumulation in subcutaneous tumors in mice [15]. Injection of these NPs in immunodeficient Swiss nude mice with implanted human prostate cancer PC3 resulted in strong and long-term fluorescent labeling of the tumor [15], but data are lacking concerning NP stability. LipImage^TM^ 815 NPs (80 nm) also accumulated in RM1 subcutaneous tumors at 24 h, but their long-term residence could not be confirmed, as tumors grew very quickly, requiring rapid euthanasia of the mice. The post-mortem analysis of individual organs excised 24 h post NP injection showed high accumulation in the liver, but the fluorescent signal in the tumor was about 4 times less, and was not clearly different from those in organs such as the kidneys, intestines or spleen. As subcutaneous tumors grew on the surface, fluorescence from NP accumulation was easily detected, and changes in the fluorescence signal could be quantified. That makes this model quite attractive, as small chemical modifications in NPs induced changes in NP accumulation in the tumor that could be easily monitored by FRI [12].

RM1 orthotopic tumors are deep in the body and thus detection of the fluorescence signal was impaired by photon absorption by the tissues. However, a deep tumor location is more favorable for fluorescence tomography, and FMT allowed for fluorescence detection and absolute in-depth quantification. Both in vivo and ex vivo imaging methods confirmed high levels of accumulation of LipImage^TM^ 815 in RM1 orthotopic tumors. Because they grow in an original microenvironment, orthotopic tumors are relevant from a physio-pathological point of view; but, as they exhibit a high level of passive NP accumulation, they behave quite differently from the clinical context.

LipImage^TM^ 815 accumulation in disseminated metastasis is low at 24 h. Combined with in-depth localization, the fluorescence signal is not detectable by in vivo fluorescence imaging. Tumor localization is made possible by BLI, and a fluorescence signal is detectable by ex vivo FRI only in organs with low background. The low level of LipImage^TM^ 815 accumulation in RM1 metastasis may be relevant for screening new strategies dedicated to circumventing EPR failures currently reported in a clinical context [16].

The choice of animal model necessary for rapid and extensive evaluation of NPs requires improvement towards a more clinically-relevant model for imaging probe evaluation. As illustrated by this study using LipImage^TM^ 815, even when using a single NP and a single cell line, non-specific accumulation clearly depends on a lot of factors, including tumor type and location. Other factors, such as tumor size, injection routes, doses and injection scheme, may further influence NP accumulation. Although it is not wholly representative of the clinical context, each tumor type may be useful for challenging NP properties.

## 4. Materials and Methods

### 4.1. Animal Handling and Tumor Generation

Animal manipulations, approved by the local ethical committee (CEEA 50) under agreement A50120196, were performed in agreement with French and European directives on the care and use of animals. B6 Albino (B6N-*Tyr^c−Brd^*/BrdCrCrl) mice (6- to 8-weeks-old) were maintained in standard conditions under a 12-h light/dark cycle with water and food provided ad libitum at the University of Bordeaux animal facilities. Manipulations were performed on anesthetized animals using 2% isoflurane (Belamont, Nicholas Piramal Limited, London, UK) in air.

Orthotopic tumors were induced by cell injection within the prostate on anesthetized mice. The skin and the abdominal muscles were incised by a short section and the seminal glands were pulled back outside the body. Cells (5 × 10^5^/10 µL per lobe) were injected in the two dorsal prostate lobes and the seminal glands returned to the abdomen. The incision was then closed with sutures. Metastasis was induced by intra-cardiac cell injection (1 × 10^5^/100 µL) in the left ventricle with ultrasound guidance on anesthetized mice. Subcutaneous tumors were generated by cell injection (2 × 10^6^/100 µL) in the posterior right leg. Before the imaging session, regions to be imaged were shaved with clippers and depilatory cream. LipImage^TM^ 815 (31.5 µM of NIR fluorophore) were injected via the tail vein. After in vivo imaging, organs were removed from euthanized mice and placed in cold phosphate-buffered saline (PBS) in a petri dish and imaged ex vivo.

### 4.2. LipImage^TM^ 815 Synthesis and Characterization

The IR780-lipid dye was first synthetized [15]. An oil premix with, respectively, 85, 255, and 65 mg of oil, Suppocire NB™ (Gattefosse S.A., Saint-Priest, France) and lecithin was prepared. IR780-lipid dye solution (10 mg/mL; 200 μL) in ethanol was poured into a 5-mL vial and mixed with the oil premix melted at 50 °C. The mixture was homogenized and the solvent was then evaporated under argon flux. After homogenization at 50 °C, the continuous aqueous phase, composed of 345 mg of Myrj^TM^ S40 (Croda Uniquema; Chocques, France) and the appropriate amount of aqueous solution (154 mM NaCl qs 2 mL), was introduced. The mixture was placed in a water bath at 50 °C and was then sonicated for 5 min using a VCX750 Ultrasonic processor (power output 190 W, 3-mm probe diameter, Sonics). LipImage^TM^ 815 solution was dialyzed against 1000 times their volume in the appropriate aqueous buffer overnight at room temperature (12 to 14,000 Da M_W_ cut off membranes, ZelluTrans, Carl Roth, France). The nanoparticle dispersion was finally sterilized by filtration through a 0.22 μm Millipore membrane. Size distribution of LipImage™ 815 was measured with a Zetasizer (Nano ZS, Malvern instrument, Worcestershire, UK) (Figure S2). The number of particles was calculated by dividing the total volume of lipids (total mass of oil, wax, lecithin and PEG assuming an overall density of 1.05 g·cm^−3^) divided by the individual volume of LipImage^TM^ 815 nanoparticles (size = 74.4 nm; Figure S2).

### 4.3. Cell Line Generation and Culture

Murine prostate cancer cell line RM1, initially obtained from Dr. T.C. Thompson (Baylor College of Medicine, Houston, TX, USA), was genetically engineered for constitutive expression of firefly luciferase (RM1-CMV/Fluc) as previously described [12], and was maintained in Dulbecco’s modified Eagle’s medium (Invitrogen, Carlsbad, CA, USA) supplemented with 10% fetal bovine serum (Invitrogen), 1% antimycotic-antibiotic mix (PSA, Invitrogen) and blasticidin (10 µg/mL, Euromedex, Souffelweyersheim, France). Cell line was maintained in a humidified 5% CO_2_ incubator at 37 °C.

### 4.4. Bioluminescence Imaging (BLI)

BLI was performed at Vivoptic (UMS 3767—Univ. Bordeaux) using Lumina LT (Perkin Elmer Inc., Boston, MA, USA). Mice received an intra-peritoneal injection of d-luciferin (2.9 mg in 100 µL PBS, Promega, Madison, WI, USA), and were anesthetized 5 min later. Bioluminescence images (1 min, 4 × 4 binning) and photographs (100 ms) were acquired successively 8 min after d-luciferin injection. Images acquisition and analysis were performed using Living Image software.

### 4.5. Fluorescence Reflectance Imaging (FRI)

FRI was performed using the Lumina LT apparatus (Perkin Elmer, Boston, MA, USA) with the 745 nm excitation filter and the 810–875 nm emission filter. Fluorescence images (1 s, 4 × 4 binning) and photographs (100 ms) were acquired successively, and analyzed using Living Image software. FRI signal is expressed as photons·s^−1^·cm^−2^·sr^−1^.

FRI was also performed using the per-operatory camera system Fluobeam^®^ (Fluoptics, Grenoble, France) at a spectral window of excitation of 780 nm and with an emission of 820 nm. The image was analyzed using Image J software.

### 4.6. Fluorescence Molecular Tomography (FMT)

Mice were imaged in a Fluorescence Molecular Tomograph (FMT^®^) 4000 (Perkin Elmer, Boston, MA, USA). Scanning was performed using the 745 channel, and the fluorescence signal was filtered with the 770–800 nm filter emission. The images were reconstructed and analyzed using the TrueQuant software.

### 4.7. Histology and Microscopic Imaging

Tumors were frozen and stored at −80 °C. Tumor slices (10 µm) were obtained, fixed with 4% paraformaldehyde (10 min, room temperature), and hematoxylin-eosin-safran staining was performed. LipImage^TM^ 815 fluorescence detection was performed using Leica DM 5500 microscope fitted with pE-100 (Ex 770 nm) Cool LED and a indocyanine (775/845 nm) filter (Leica Microsystems, Wetzlar, Germany).

## Supplementary Materials

Supplementary materials can be found at www.mdpi.com/1422-0067/18/12/2584/s1.
