# Supplementary material for: In Vivo Imaging of Prostate Cancer Tumors and Metastasis Using Non-Specific Fluorescent Nanoparticles in Mice
Source: Int J Mol Sci. 2017 Dec 1;18(12):2584. doi: 10.3390/ijms18122584 (PMC5751187; doi:10.3390/ijms18122584)
Supplement: Supplementary file 1 [file ijms-18-02584-s001.zip › Supplementary caption.pdf]

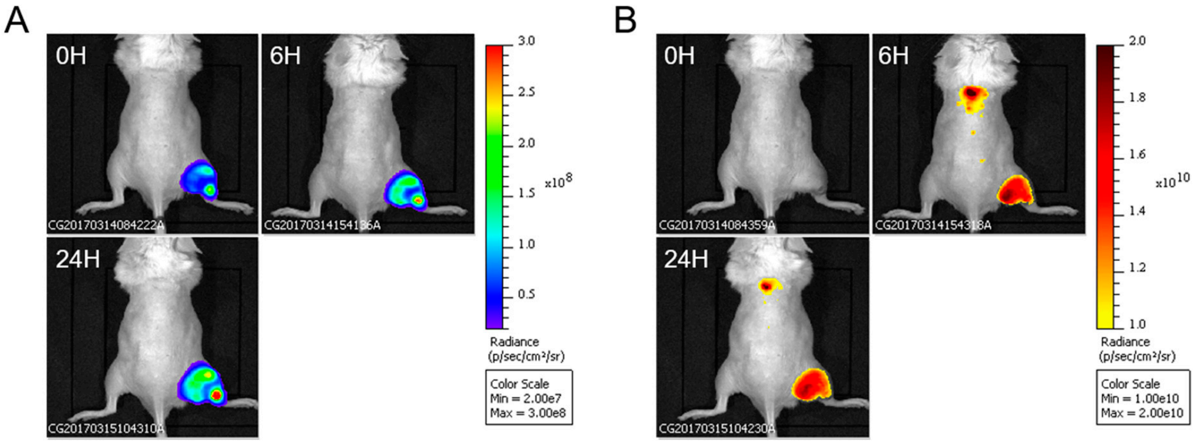

Supplementary Figure S1 Bioluminescence and Fluorescence imaging of a representative mouse bearing subcutaneous tumor. BLI (Panel A) and FRI (Panel B) before LipImage^{TM} 815 injection (0H), 6 hours (6H) and 24 hours (24H) after LipImage^{TM} 815 injection.

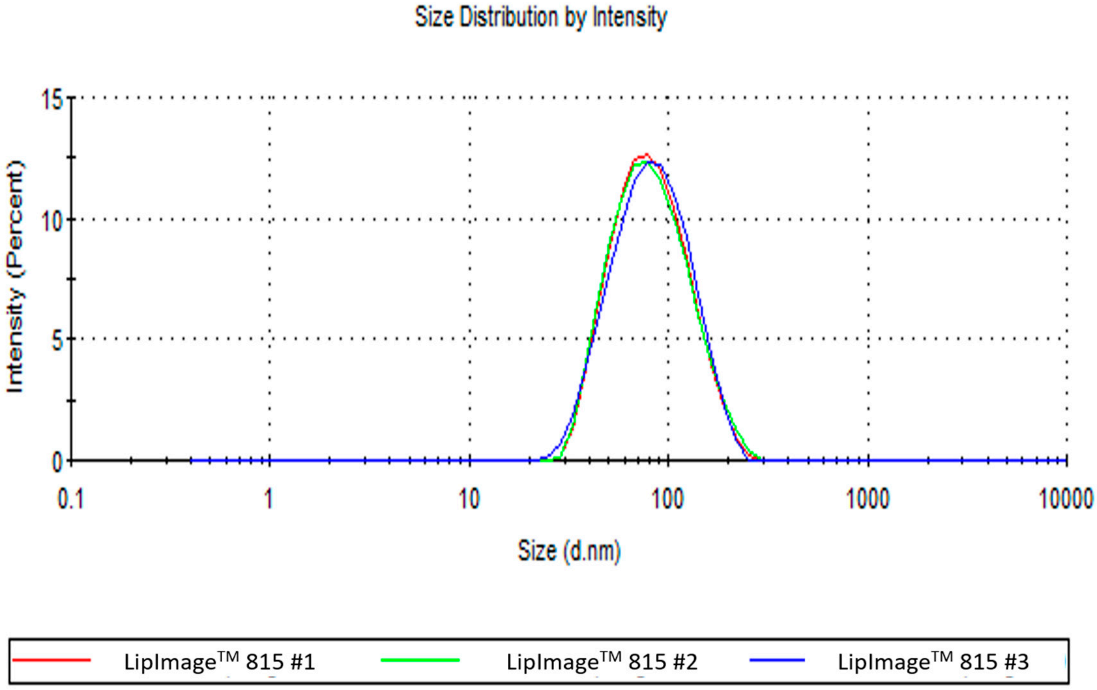

Supplementary Figure S2 Size distribution of LipImage^{TM} 815 F80 nanoparticules . Z average of 3 measures is 74.4 nm and polydispersity index (PDI) is 0.161 using a Zetasizer Nano ZS equipment (MALVERN).

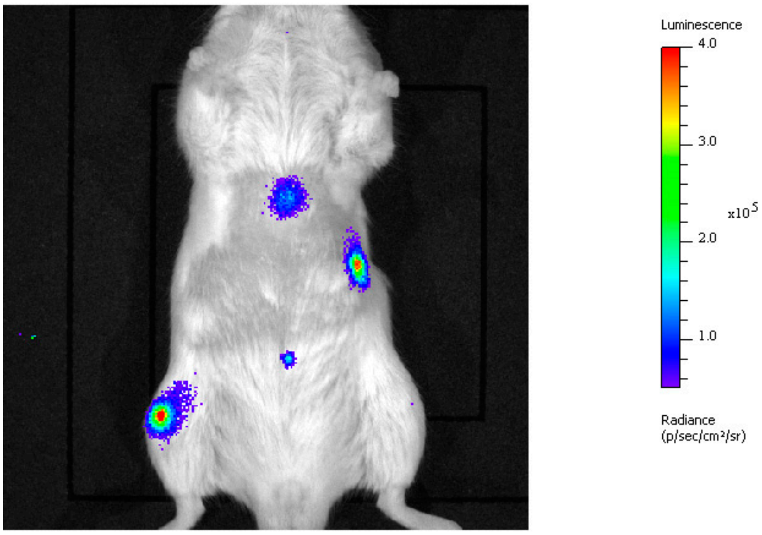

Supplementary Figure S3: Bioluminescence imaging of a representative mouse bearing metastasis 7 days after cells injection.

Supplementary caption:

Video 1: Video of FRI of a mouse after intravenous injection of LipImageTM 815.

Video 2: Video of echography-guided intra-cardiac injection of 100 000 RM1 cells in anesthetized mouse.
